# The safety and efficacy of quadrivalent live attenuated influenza vaccine in Japanese children aged 2‐18 years: Results of two phase 3 studies

**DOI:** 10.1111/irv.12555

**Published:** 2018-04-10

**Authors:** Raburn M. Mallory, Jing Yu, Sachiko Kameo, Michio Tanaka, Ki Rito, Yohji Itoh, Filip Dubovsky

**Affiliations:** ^1^ MedImmune Gaithersburg MD USA; ^2^ AstraZeneca Japan Osaka Japan; ^3^ Formerly of AstraZeneca Japan Tokyo Japan

**Keywords:** Japan, LAIV, pediatric, seasonal influenza

## Abstract

**Background:**

Quadrivalent live attenuated influenza vaccine (Q/LAIV) has not been assessed in Japanese children.

**Objectives:**

Evaluate safety and efficacy of Q/LAIV in Japanese children.

**Patients/methods:**

Two phase 3 studies were conducted in the 2014‐2015 influenza season. Study 1 was an open‐label, uncontrolled single arm, multicenter study of Q/LAIV safety in subjects aged 2‐6 years. Study 2 was a randomized, double‐blind, placebo‐controlled multicenter study of Q/LAIV safety and efficacy; subjects aged 7‐18 years were randomized 2:1 to receive Q/LAIV or placebo. Primary efficacy endpoint was laboratory‐confirmed symptomatic influenza infection caused by vaccine‐matched strains; secondary endpoint evaluated efficacy against all strains regardless of match. Both studies reported solicited symptoms, adverse events (AEs), and serious AEs.

**Results:**

In Study 1, 100 subjects received Q/LAIV. In Study 2, 1301 subjects received Q/LAIV (n = 868) or placebo (n = 433). Treatment‐emergent AEs occurred in 42% of subjects in Study 1, and in 24.3% of subjects in the Q/LAIV arm and in 25.9% of subjects in the placebo arm in Study 2. In Study 2, a single infection by a vaccine‐matched strain was reported in the placebo arm, resulting in a vaccine efficacy estimate of 100% (95% CI: −1875.3, 100.0); efficacy for all strains regardless of match to the vaccine was 27.5% (95% CI: 7.4, 43.0).

**Conclusions:**

Quadrivalent live attenuated influenza vaccine did not meet its primary efficacy endpoint as only a single infection by a vaccine‐matched strain was detected; however, efficacy for the secondary endpoint, all strains regardless of match, was achieved. Q/LAIV was generally well tolerated in the Japanese pediatric population.

## 1 INTRODUCTION

Seasonal influenza affects an estimated 5%‐10% of adults and 20%‐30% of children worldwide.^1^ In Japan alone, it is estimated that over 10 million people are infected with the influenza virus annually.^2^ The global burden of influenza is substantial, and the World Health Organization estimates that influenza causes 3‐5 million cases of severe illness and 300 000‐500 000 deaths annually.^3^ Children are at high risk of developing serious complications from influenza such as pneumonia and secondary bacterial infections,^4^ and on a global level, influenza is associated with 10% of respiratory hospitalizations in children under 18 years old.^5^

Influenza prevention is particularly important in those under 18 years of age, as children have the highest influenza attack rates^6^ and are key transmitters of influenza in the community as they can shed a greater amount of virus for longer periods of time in comparison with adults.^7^, ^8^ In Japan, children have traditionally been vaccinated against influenza using trivalent inactivated influenza vaccines (TIVs) consisting of 3 influenza strains: two A strains and one B strain. However, 2 antigenically distinct lineages of influenza B (Victoria and Yamagata) commonly cocirculate in an influenza season. As Global Health Authorities have had limited success in selecting the B strain for trivalent vaccines that match the predominant circulating B strains,^9^ it has been suggested that a switch from TIV to a quadrivalent vaccine could reduce the incidence of influenza infection.^10^

The intranasally administered quadrivalent live attenuated influenza vaccine (Q/LAIV) contains 4 virus strains: two type A strains (A/H1N1 and A/H3N2), and two type B strains: one from each of the Victoria and Yamagata lineages. Q/LAIV is currently used in North America and several countries in Europe, including the United Kingdom, which has implemented a national pediatric vaccination program using Q/LAIV.^11^ Although multiple studies have documented the safety and efficacy of Q/LAIV among children in countries outside Japan,^12^, ^13^, ^14^ Q/LAIV has not previously been assessed in Japanese children.

Here, we report the results of two phase 3 clinical trials of Q/LAIV in Japan: an open‐label study of the safety and tolerability of Q/LAIV among Japanese children aged 2‐6 years, and a randomized, double‐blind, placebo‐controlled study of the safety and efficacy of Q/LAIV among Japanese children aged 7‐18 years.

## 2 METHODS

### 2.1 Study design

The first study (Study 1) was a phase 3 open‐label, uncontrolled, multicenter study (ClinicalTrials.gov identifier: NCT02269488), which was conducted across 3 centers in Japan during the 2014‐2015 influenza season to evaluate the safety and tolerability of Q/LAIV among Japanese children aged 2‐6 years. The second study (Study 2) was a phase 3 randomized, double‐blind, placebo‐controlled multicenter study (ClinicalTrials.gov identifier: NCT02269475), which was conducted across 49 centers in Japan during the same influenza season to evaluate the safety and efficacy of Q/LAIV among Japanese children aged 7‐18 years.

Both studies were performed in accordance with the principles of the Declaration of Helsinki, which are consistent with the International Conference on Harmonization (ICH)/Good Clinical Practice (GCP) and applicable regulatory requirements, and the AstraZeneca policy on Bioethics and Human Biological Samples. Both protocols were approved by the Institutional Review Board (IRB) of each study center, and all participants and/or legal representatives provided written informed consent.

### 2.2 Vaccine

Each dose of LAIV contained 10^7.0±0.5^ FFU of each of the 4 cold‐adapted, attenuated, temperature‐sensitive, 6:2 reassortant influenza strains (A/H1N1; A/California/7/2009, A/H3N2; A/Texas/50/2012; B/Brisbane/60/2008 [Victoria lineage], and B/Massachusetts/2/2012 [Yamagata lineage]). The clinical trial material used in the study was tested for potency, at the time it was released for clinical use using the same FDA/EMA approved methods that are used to release the commercially distributed vaccine. To ensure that the vaccine continued to meet its potency requirements, the vaccine was shipped and distributed to sites in Japan using validated shipping methods.

### 2.3 Patients

#### 2.3.1 Study 1

The study planned to enroll approximately 100 subjects aged 2‐6 years to assess the safety and tolerability of Q/LAIV. All subjects received an initial intranasal administration of 0.2 mL (0.1 mL per nostril) of vaccine, while subjects not previously vaccinated against seasonal influenza received a second dose after an interval of at least 4 weeks. Healthy subjects and those with chronic underlying medical conditions including mild‐to‐moderate asthma were included, provided the subject had not been hospitalized in the previous year. Subjects with asthma were included in this study and in Study 2 (below) based on the demonstrated safety and efficacy of LAIV in this population,^15^ and as these subjects are included in the indicated populations who can receive Q/LAIV in Europe and Canada.^16^, ^17^

#### 2.3.2 Study 2

This double‐blind study planned to enroll 1008 subjects aged 7‐18 years to assess the safety and efficacy of Q/LAIV. Subjects were randomized (2:1) to receive Q/LAIV or placebo via intranasal administration. Subjects received an intranasal administration of 0.2 mL (0.1 mL per nostril). Subjects aged 7‐8 years not previously vaccinated against seasonal influenza received a second dose after an interval of at least 4 weeks. Consistent with Study 1, healthy subjects and those with chronic underlying medical conditions were included, provided hospitalization was not required in the previous year.

#### 2.3.3 Determination of analysis set

In both Study 1 and Study 2, safety was assessed using the safety population, which included all subjects who received any amount of investigational product. For the safety population, subjects were analyzed per the treatment they received for the first dose. The per‐protocol (PP) population was used for efficacy assessments in Study 2, and included all subjects who had no important protocol deviations, received the first and second dose of study vaccine or placebo per protocol, and were followed up for qualifying symptoms for influenza until the end of the influenza season.

### 2.4 Endpoints

#### 2.4.1 Safety and tolerability

In both Study 1 and Study 2, solicited symptoms were collected for 14 days post‐vaccination and included the following: fever ≥100.4°F (38.0°C) by any route, runny/stuffy nose, sore throat, cough, headache, generalized muscle aches, decreased activity level or tiredness/weakness, and decreased appetite. Adverse events (AEs), including treatment‐emergent adverse events (TEAEs), were monitored for 28 days post‐vaccination, and if patients received 2 doses of vaccine, they were monitored through the last dose. Serious adverse events (SAEs) were monitored from the time of informed consent through the last study contact with the subject.

#### 2.4.2 Efficacy

In Study 2, the primary efficacy endpoint was laboratory‐confirmed symptomatic influenza infection (modified influenza‐like illness per the Centers for Disease Control [mCDC‐ILI]) caused by any community‐acquired wild‐type strains matched to the vaccine. The secondary efficacy endpoint was laboratory‐confirmed symptomatic influenza infection (mCDC‐ILI) caused by any community‐acquired wild‐type strains regardless of match to the vaccine. Modified CDC‐ILI was defined as increased temperature ≥100°F (37.8°C) (oral or equivalent) plus the presence of cough, sore throat, or runny nose/nasal congestion occurring on the same or consecutive days. Nasal swabs were evaluated for influenza using a polymerase chain reaction‐based test, and genotyping, subtyping, and sequencing were performed. Isolates were categorized as vaccine‐like (matched) or non‐vaccine‐like (mismatched) using genetic sequence alignment to reference strains for which a determination of match or mismatch was publicly available from the US CDC or other World Health Organization collaborating centers. For both efficacy endpoints, the influenza infection rate in Q/LAIV recipients was compared to that in placebo recipients during the influenza surveillance period and at least 14 days after the last administered vaccination.

### 2.5 Statistical analyses

Categorical data were summarized by the number and percentage of subjects falling within each category, and continuous variables were summarized using descriptive statistics including mean, standard error or deviation, median, minimum, and maximum.

The primary and secondary efficacy endpoints evaluated vaccine efficacy, that is, the risk reduction of influenza infection in Q/LAIV recipients compared to placebo recipients calculated as one minus the ratio of the infection rates. Statistical comparison was made by constructing 2‐sided 95% confidence intervals (CI) for the efficacy estimate, and the CI was estimated by an exact conditional method conditioning on the total number of cases, which followed a Poisson assumption. If the lower bound of the 95% CI of vaccine efficacy was >0%, then the efficacy of Q/LAIV was demonstrated.

In Study 1 and Study 2, all safety evaluations were descriptive in nature, and in Study 2, safety evaluations were provided by the dose number and by treatment group. AEs and TEAEs were summarized by system organ class (SOC) and preferred term (PT) using the Medical Dictionary for Regulatory Activities (MedDRA) version 17.1.

In both studies, statistical analysis was performed by Quintiles Transnational Japan K.K. in Japan. All analyses were conducted using Statistical Analysis System (SAS) version 9.2.

## 3 RESULTS

### 3.1 Study subjects: Study 1

All 100 enrolled Japanese children aged 2‐6 years completed the study and were included in the safety analysis. The mean (SD) age of subjects was 4.2 (±1.4) years and male subjects accounted for 45.0% (45/100 subjects) of the population (Table 1). Of the 100 subjects, 25.0% (25/100) had preexisting medical conditions; the most frequent conditions were asthma (14%), allergic rhinitis (5%), and dermatitis (5%). A majority of the 100 subjects enrolled had previously received an influenza vaccination (94.0%, 94/100), and thus received a single dose of Q/LAIV. The remaining 6.0% (6/100) of subjects who had not been previously vaccinated received 2 doses of vaccine.

**Table 1:** Study 1 subject demographics (safety population, aged 2**‐**6 y)

Characteristics	Q/LAIV (n = 100)
Age (y), mean (SD)	4.2 (1.4)
Male, n (%)	45 (45.0)
Number of doses of study vaccine received, n (%)	
One	94 (94.0)
Two	6 (6.0)
Preexisting medical condition	
Yes, n (%)	25 (25.0)

Q/LAIV, quadrivalent live attenuated influenza vaccine; SD, standard deviation.

### 3.2 Study subjects: Study 2

A total of 1369 Japanese children aged 7‐18 years were enrolled in Study 2. Of these, 1301 subjects were randomized to Q/LAIV (868) or placebo (433) with a 2:1 ratio at 49 study centers in Japan. Sixty‐eight subjects were not randomized, primarily because randomization had closed after they were screened. All 1301 randomized subjects were included in the safety analysis.

The mean (SD) age of subjects was 11.0 (±3.0) years in the Q/LAIV group and 10.8 (±2.8) years in the placebo group (Table 2). 32.5% (282/868 subjects) in the Q/LAIV group and 28.4% (123/433 subjects) in the placebo group had preexisting medical conditions. The most frequent preexisting conditions in the Q/LAIV group and placebo group were allergic rhinitis (16.6% vs 15.5%), asthma (5.5% vs 4.8%), and seasonal allergy (3.8% vs 3.7%), respectively. A history of prior vaccination was reported in 90.8% (788/868) of subjects in the Q/LAIV group and 89.1% (386/433) of subjects in the placebo group. Most subjects (1297) completed the study, and 1279 subjects were included in the PP population.

**Table 2:** Study 2 subject demographics (safety population; aged 7**‐**18 y)

Characteristics	Q/LAIV (n = 868)	Placebo (n = 433)	Total (n = 1301)
Age (y), mean (SD)	11.0 (3.0)	10.8 (2.8)	10.9 (2.9)
Male, n (%)	460 (53.0)	207 (47.8)	667 (51.3)
Number of doses of study vaccine received, n (%)			
One	841 (96.9)	421 (97.2)	1262 (97.0)
Two	27 (3.1)	12 (2.8)	39 (3.0)
Preexisting medical condition			
Yes, n (%)	282 (32.5)	123 (28.4)	405 (31.1)

Q/LAIV, quadrivalent live attenuated influenza vaccine; SD, standard deviation.

### 3.3 Safety and tolerability

#### 3.3.1 Solicited symptoms

In Study 1, the overall incidence of solicited symptoms was 57.0% (57/100 subjects). The most common solicited symptoms (≥5%) were runny/stuffy nose (51.0%), cough (34.0%), fever ≥100.4°F (38.0°C) (10.0%), and sore throat (7.0%; Table 3). In Study 2, the overall incidence of subjects with solicited symptoms was 41.7% (362/868) in the Q/LAIV group and 40.6% (176/433 subjects) in the placebo group. There were no notable differences in the incidence of solicited symptoms between the placebo and active arm of the study. The most common solicited symptoms (≥5% in either group) were runny/stuffy nose (33.2%), cough (12.4%), sore throat (10.1%), and headache (9.6%) in the Q/LAIV group, and runny/stuffy nose (27.7%), cough (15.9%), sore throat (14.1%), and headache (8.5%) in the placebo group (Table 4). The incidence rates for individual symptoms were similar between groups, with the exception of a slightly higher incidence rate of runny/stuffy nose in the Q/LAIV group (rate difference: 5.5%, 95% CI: 0.1, 10.6).

**Table 3:** Solicited symptoms observed among the safety population (Study 1; aged 2**‐**6 y)

	Q/LAIV (n = 100)
Number of subjects with any solicited symptoms, n (%)^a^	57 (57.0)
Runny/stuffy nose	51 (51.0)
Cough	34 (34.0)
Temperature (Fever ≥ 100.4°F [38.0°C] by any route)	10 (10.0)
Sore throat	7 (7.0)
Headache	3 (3.0)
Decreased activity level (lethargy) OR tiredness/weaknesses	3 (3.0)
Decreased appetite	2 (2.0)
Generalized muscle aches	1 (1.0)

Q/LAIV, quadrivalent live attenuated influenza vaccine.

a A subject with at least 1 solicited symptom was counted once.

**Table 4:** Solicited symptoms observed among the safety population (Study 2; aged 7**‐**18 y)

	Q/LAIV (n = 868)	Placebo (n = 433)	Rate difference point estimate (95% CI)
Number of subjects with any solicited symptoms, n (%)^a^	362 (41.7)	176 (40.6)	1.1 (−4.7, 6.7)
Runny/stuffy nose	288 (33.2)	120 (27.7)	5.5 (0.1, 10.6)
Cough	108 (12.4)	69 (15.9)	−3.5 (−7.8, 0.4)
Sore throat	88 (10.1)	61 (14.1)	−3.9 (−8.0, −0.3)
Headache	83 (9.6)	37 (8.5)	1.0 (−2.5, 4.2)
Decreased activity level (lethargy) OR tiredness/weaknesses	37 (4.3)	11 (2.5)	1.7 (−0.6, 3.7)
Temperature (fever ≥ 100.4°F [38.0°C] by any route)	26 (3.0)	12 (2.8)	0.2 (−2.0, 2.1)
Decreased appetite	20 (2.3)	10 (2.3)	0.0 (−2.1, 1.6)
Generalized muscle aches	9 (1.0)	2 (0.5)	0.6 (−0.8, 1.6)

CI, confidence interval; Q/LAIV, quadrivalent live attenuated influenza vaccine.

a A subject with at least 1 solicited symptom was counted once.

#### 3.3.2 Adverse events

In Study 1, during the 28 days after the first vaccination, TEAEs were noted in 42.0% (42/100) of subjects. Two of 6 subjects (33.3%) without previous influenza vaccination who received 2 doses of the vaccine experienced TEAEs during the 28 days after the second vaccination, and all TEAEs were mild in intensity (Table 5). The only TEAE reported at a rate ≥5% was nasopharyngitis (13.0%), and no febrile convulsions were reported.

**Table 5:** Overview of treatment‐emergent adverse events (TEAEs) among the safety population (Study 1; aged 2**‐**6 y)

Description	After 1st dose (n = 100)	After 2nd dose (n = 6)
Number of subjects with		
At least 1 TEAE, n (%)	42 (42.0)	2 (33.3)
At least 1 TEAE related to the IP, n (%)^a^	1 (1.0)	0 (0.0)
At least 1 treatment‐emergent SAE, n (%)	0 (0.0)	0 (0.0)
At least 1 treatment‐emergent SAE related to the IP, n (%)	0 (0.0)	0 (0.0)
At least 1 SAE, n (%)	0 (0.0)	0 (0.0)
A TEAE leading to study discontinuation, n (%)	0 (0.0)	0 (0.0)
A TEAE leading to death, n (%)	0 (0.0)	0 (0.0)
Intensity of TEAE		
Mild, n (%)	42 (42.0)	2 (33.3)
Moderate, n (%)	0 (0.0)	0 (0.0)
Severe, n (%)	0 (0.0)	0 (0.0)

IP, investigational product; Q/LAIV, quadrivalent live attenuated influenza vaccine; SAE, serious adverse event.

a Two TEAEs, abdominal pain, and diarrhea were reported after the first dose by 1 subject.

During the study, no SAEs were reported, and no discontinuations of the investigational product (IP) due to an AE were reported. Two TEAEs, abdominal pain, and diarrhea were reported in one subject after the first dose and were considered by the investigator to be related to the IP. One significant AE was reported as follows: a non‐serious case of erythema multiforme occurred in a 6‐year‐old male subject with a history of previous influenza vaccination and without any medical history at day 26 after Q/LAIV administration. The TEAE was confirmed to be mild in intensity, not related to vaccination in terms of causality and resolved by day 51.

In Study 2, the incidence of TEAEs was 24.3% (211/868) in the Q/LAIV group and 25.9% (112/433) in the placebo group, with no obvious differences in the profile of TEAEs between the 2 groups. The incidence of mild TEAEs was 21.4% (186/868) in the Q/LAIV group and 22.9% (99/433) in the placebo group, while moderate TEAEs were reported in 2.9% (25/868) of subjects in the Q/LAIV group and 3.0% (13/433) of subjects in the placebo group. No severe TEAEs were noted in either the Q/LAIV or placebo group (Table 6). The only TEAE reported at a rate ≥5% was nasopharyngitis: 8.1% (70/868) of subjects in the Q/LAIV group and 8.3% (36/433) of subjects in the placebo group, and no febrile convulsions were reported. SAEs were reported in 3 (0.3%) subjects in the Q/LAIV group and 3 (0.7%) subjects in the placebo group. The SAEs reported in the Q/LAIV group were peritonsillar abscess, convulsion, and appendicitis, and they occurred at 112, 115, and 134 days post‐vaccination, respectively, while SAEs reported in the placebo group were pneumonia, osteochondrosis, and hydronephrosis. All SAEs were considered to be unrelated to receipt of the IP.

**Table 6:** Overview of treatment‐emergent adverse events (TEAEs) among the safety population (Study 2; aged 7**‐**18 y)

Description	Q/LAIV (n = 868)	Placebo (n = 433)
Number of subjects with		
At least 1 TEAE^a^, n (%)	211 (24.3)	112 (25.9)
At least 1 TEAE related to the IP^a^, n (%)	4 (0.5)	1 (0.2)
At least 1 treatment‐emergent SAE^a^, n (%)	0 (0.0)	1 (0.2)
At least 1 treatment‐emergent SAE related to IP^a^, n (%)	0 (0.0)	0 (0.0)
At least 1 SAE^b^^,^ c, n (%)	3 (0.3)	3 (0.7)
A TEAE leading to study discontinuation, n (%)	0 (0.0)	0 (0.0)
A TEAE leading to death^a^, n (%)	0 (0.0)	0 (0.0)
Intensity of TEAE		
Mild, n (%)	186 (21.4)	99 (22.9)
Moderate, n (%)	25 (2.9)	13 (3.0)
Severe, n (%)	0 (0.0)	0 (0.0)

IP, investigational product, Q/LAIV, quadrivalent live attenuated influenza vaccine; SAE, serious adverse event.

a Defined as occurring from Q/LAIV administration through 28 d post‐vaccination.

b Defined as occurring from informed consent through the end of the study.

c All SAEs were considered to be unrelated to receipt of the IP.

### 3.4 Efficacy

In Study 2, in the PP population, swab samples for genotyping, subtyping, and sequencing were collected from 532 subjects (62.7%) in the MEDI3250 group and from 299 subjects (69.5%) in the placebo group through the end of the 2014‐2015 influenza season. Swab samples were collected at least 14 days after vaccination for 513 subjects (60.4%) and 288 subjects (67.0%), respectively. Among the PP population, influenza caused by vaccine‐matched strains based on the definition of mCDC‐ILI was reported in one of 430 subjects (0.2%) in the placebo group. The subject was infected with a B/Yamagata lineage strain, which resulted in a vaccine efficacy estimate of 100% (95% CI: −1875.3, 100.0; Table 7).

**Table 7:** Vaccine efficacy against based on the definition of modified influenza‐like illness per the Centers for Disease Control (per‐protocol population, Study 2; aged 7**‐**18 y)

	Subjects with influenza infection		Vaccine efficacy (%)
	Q/LAIV (n = 849)	Placebo (n = 430)	Point estimate (95% CI)
Influenza due to matched strains^a^, n (%)	0 (0.0)	1 (0.2)	100.0 (−1875.3, 100.0)
Influenza due to all strains regardless of match^b^, n (%)	169 (19.9)	118 (27.4)	27.5 (7.4, 43.0)
Influenza due to all H1N1 strains regardless of match, n (%)	0 (0.0)	0 (0.0)	—
Influenza due to mismatched H1N1 strains, n (%)	0 (0.0)	0 (0.0)	—
Influenza due to all H3N2 strains regardless of match, n (%)	165 (19.4)	112 (26.0)	25.4 (4.3, 41.7)
Influenza due to mismatched H3N2 strains, n (%)	163 (19.2)	112 (26.0)	26.3 (5.4, 42.4)
Influenza due to all B strains regardless of match, n (%)	4 (0.5)	6 (1.4)	66.2 (−42.4, 93.0)
Influenza due to mismatched B strains, n (%)	3 (0.4)	3 (0.7)	49.4 (−278.1, 93.2)

CI, confidence interval (2‐sided); Q/LAIV, quadrivalent live attenuated influenza vaccine.

a Vaccine‐matched strains were defined as isolates classified as vaccine‐like by sequencing for samples collected at least 14 d after the last required vaccination through the end of the 2014‐2015 influenza season.

b Influenza caused by any strain was defined as isolates classified as *wild type* or a mixture of *wild type* and *cold‐adapted* by genotyping. Efficacy for strains regardless of match refers to efficacy for both matched and mismatched strains.

Among the PP population, the incidence of influenza caused by any strain regardless of match to the vaccine based on the definition of mCDC‐ILI was 19.9% (169/849 subjects) in the Q/LAIV group and 27.4% (118/430) in the placebo group. The estimate of vaccine efficacy for any strain regardless of match to the vaccine was 27.5% (95% CI: 7.4, 43.0) (Table 7). Vaccine efficacy for all strains regardless of match by age group was 22.9% (95% CI −19.0, 49.5) for children aged 7‐8 years and 30.2% (95% CI 5.6, 48.1) for children aged 9‐18 years.

The vast majority strains (275/287) that circulated during the study were H3N2 strains that were significantly mismatched to the vaccine H3N2 strain. For these strains, the vaccine had an efficacy estimate of 26.3% (95% CI: 5.4, 42.4) (Table 7).

## 4 DISCUSSION

Children are at high risk of influenza infection compared with other age groups and play an important role in spreading influenza in the community.^8^ The benefit of vaccinating children against influenza is twofold: a direct reduction of morbidity and mortality in the pediatric population, which can also extend to the community around them through reduced rates of secondary transmission.^18^

In these first studies of the safety and efficacy of Q/LAIV in Japanese children, Q/LAIV was generally well tolerated, and the incidence of solicited symptoms and adverse events was similar to those observed in studies conducted outside of Japan.^19^, ^20^ The safety of trivalent LAIV in children 2 years of age and older has been well established in over 70 studies enrolling more than 140 000 subjects^19^ and through company‐sponsored post‐marketing safety surveillance of the more than 60 million doses distributed since initial approval of the vaccine in 2003. Similarly, the safety of the quadrivalent formulation of the vaccine in children 2 years of age and older has been well established through studies enrolling over 60 000 subjects^20^, ^21^ and through company‐sponsored post‐marketing safety surveillance of the more than 50 million doses distributed since initial approval of quadrivalent LAIV in 2012.

In terms of efficacy, Q/LAIV did not meet its primary endpoint of efficacy against matched strains, as only a single vaccine‐matched strain from the B/Yamagata lineage was detected in the placebo group. The majority of influenza isolates detected during the study were mismatched A/H3N2 strains. However, the secondary endpoint of efficacy against any influenza strains regardless of match was met with statistically significant vaccine effectiveness of 27.5% (95% CI: 7.4, 43.0).

Similar to the experience in other countries, the 2014‐2015 influenza season in Japan was characterized by the circulation of influenza A/H3N2 strains that were significantly mismatched (eightfold to 32‐fold antigenic variation by ferret hemagglutination inhibition [HAI] antibody titers) to the strain selected by the World Health Organization and local health authorities for inclusion in both live attenuated and inactivated vaccines.^22^, ^23^ While a higher level of efficacy (86%) was reported for LAIV against mismatched strains in a previous placebo‐controlled study,^24^ it is worth noting that the degree of antigenic mismatch between the H3N2 strains in the study was small, fourfold by ferret HAI titers.^25^ The low level of vaccine efficacy observed in this study is consistent with LAIV efficacy reported in other randomized trials in which significantly mismatched (>8‐fold by ferret HAI titers) A/H3N2 strains circulated. In these studies, vaccine efficacy estimates compared to placebo for significantly mismatched H3N2 strains were 18% and 31%.^26^, ^27^ The efficacy estimates presented in this study are also consistent with those observed in a US‐based test‐negative case‐controlled effectiveness study conducted over the same 2014‐2015 season, in which the estimate of LAIV effectiveness against H3N2 strains was 24% (95% CI: −14, 49).^28^ The findings are also in agreement with the results observed in a 2014‐2015 UK‐based effectiveness study among children under 18 years of age, in which the effectiveness of LAIV against H3N2 strains was estimated at 35.0% (95% CI: −29.9, 67.5).^29^

While only a small number of B strains circulated during the study, the point estimate for LAIV efficacy against B strains (66.2%) was also consistent with estimates from studies conducted in the United States during the same season, which reported point estimates for LAIV effectiveness of 66% and 86%.^28^ In the previously mentioned UK effectiveness study, the point estimate of LAIV effectiveness against B strains was 100%.

Overall, it is likely that the substantial mismatch between the Q/LAIV A/H3N2 strain in the 2014‐2015 vaccine and the circulating A/H3N2 strains accounted for the low efficacy of the Q/LAIV in this study. Low efficacy as a result of vaccine mismatch also affected inactivated influenza vaccines in 2014‐2015. The Canadian Sentinel Physician Surveillance Network found little or no vaccine protection with LAIV or TIV during the 2014‐2015 season due to the circulation of mismatched A/H3N2 strains.^30^ In the UK effectiveness study from the same season, the inactivated influenza vaccine did not appear to be effective against H3N2 strains among children under 18 years of age (efficacy estimate of −73.2%, 95% CI: −456.9, 46.2).^29^ A similar test‐negative outpatient study conducted during the 2014‐2015 season in Japan in children 6 months through 15 years of age yielded an effectiveness estimate of 37% (95% CI: 27, 45) for the trivalent inactivated vaccine against influenza A strains.^31^

## 5 CONCLUSIONS

The two phase 3 studies reported here provide the first assessment of the safety and efficacy of Q/LAIV in Japanese children. Q/LAIV was found to be generally well tolerated in the Japanese pediatric population 2 through 18 years of age, and the safety profile was comparable to that observed in studies conducted outside of Japan. Observed efficacy was low, due to the predominance of circulating strains that were highly mismatched to the vaccine during the 2014‐2015 season. Consequently, the study did not meet its primary efficacy endpoint, efficacy against matched strains, as only a single vaccine‐matched strain was reported during the study. However, the secondary endpoint of efficacy against all strains regardless of match was met, and the results were consistent with LAIV efficacy observed in previous randomized trials in which significantly mismatched A/H3N2 strains circulated.

## CONFLICT OF INTEREST

Raburn Mallory, Jing Yu, and Filip Dubovsky are employees and shareholders of MedImmune. Sachiko Kameo, Michio Tanaka, and Yohji Itoh are employees and shareholders of AstraZeneca Japan. Ki Rito was an employee of AstraZeneca during this study.
